# Usefulness of dilated blood vessels in the tumor periphery for assessing the invasion depth of small-sized depressed colorectal cancer

**DOI:** 10.1097/MD.0000000000003913

**Published:** 2016-06-24

**Authors:** Rintaro Hashimoto, Tomoki Matsuda, Hidetaka Hamamoto, Hajime Yamaoka, Masato Nakahori, Akimichi Chonan

**Affiliations:** Department of Gastroenterology, Sendai Kousei Hospital, Miyagi, Japan.

**Keywords:** chromoendoscopy, colonoscopy, colorectal cancer, endoscopic resection, sensitivity, specificity

## Abstract

The relationship between dilated blood vessels in the tumor periphery and the tumor invasion depth is unclear. Therefore, the present study aimed to clarify the relationship between dilated blood vessels and the invasion depth of small-sized (<30 mm) colorectal cancer (CRC), and its implications on endoscopic treatment.

We performed a single-arm observational study of the diagnostic accuracy of the existence of dilated vessels in the tumor periphery of CRC lesions as an indicator of submucosal deep (SM-d, ≥1000 μm) carcinomas. Lesions were classified into two groups based on the existence of dilated vessels by two experienced endoscopists. The clinicopathological features, invasion depth, and lymphovascular invasion/poorly differentiated clusters were analyzed in all resected specimens.

Four hundred and two consecutive small-sized CRC lesions were included. The dilated vessels were observed in 96/402 (24%) lesions, and most of them (93/96) were found in depressed lesions. In depressed lesions, the histopathological diagnosis of the dilated vessels group showed SM-d or deeper invasion in 84/93 (90%) cases, whereas 3/20 (15%) had SM-d invasion in the nondilated vessels group (*P* < 0.001). When the dilated vessels were used as an indicator of SM-d or deeper invasion in depressed lesions, the sensitivity was 95.6%, specificity was 66.7%, and accuracy was 90.2%. No correlation was observed between the existence of dilated vessels and the lesion site, lesion diameter, and lymphovascular invasion/poorly differentiated cluster.

The existence of dilated blood vessels in the tumor periphery suggests SM-d or deeper invasion in depressed lesions.

## 1 Introduction

It is very important to accurately estimate the depth of invasion of early stage colorectal cancer (CRC) to make proper therapeutic decisions because patients with intramucosal carcinoma and submucosal invasive carcinoma with an invasion depth of less than 1000 μm can be cured by endoscopic resection.^[1]^ Chromoendoscopy, using Kudo and Tsuruta's pit pattern classification, is a useful tool for making the differential diagnosis of colorectal tumors, including submucosal carcinoma.^[2,3]^ Magnifying chromoendoscopy has been widely demonstrated to be effective in differentiating between colorectal neoplastic and non-neoplastic polyps, and assessing the invasion depth by using pit pattern analysis.^[4]^ Some studies have shown the effectiveness of narrow-band imaging (NBI) magnifying endoscopy for determining the invasion depth.^[5]^ Recently, the Narrow-band Imaging International Colorectal Endoscopic classification, which requires diagnoses of the vascular pattern and surface pattern, has also been used.^[6,7]^ As vascular diagnosis is not included in the diagnostic criteria for pathological grading, its relationship with the depth of tumor invasion is indirect. For colorectal intratumoral vessels, as the pathological grading increases, a larger blood vessel diameter and varying density and irregularity on a scale are observed with the depth of tumor invasion.^[8]^ However, the relationship between blood vessels in the tumor periphery and the tumor invasion depth is unclear.

## 2 Methods

### 2.1 Study design

This study was a single-arm observational study of diagnostic accuracy according to the Standards for the Reporting of Diagnostic Accuracy Studies initiative,^[9]^ which was conducted at Sendai Kousei Hospital, a tertiary referral hospital. The aim of this study was to clarify the relationship between the existence of dilated blood vessels in the tumor periphery and the invasion depth of small-sized CRC. The protocol of this retrospective study was approved by our Institutional Review Board.

### 2.2 Patients

This study included consecutive endoscopically or surgically resected 774 CRC lesions, of which the invasion depth was pathologically diagnosed as intramucosal (M), submucosal (SM), or muscularis propria (MP), in 652 patients at Sendai Kousei Hospital between January 2012 and November 2014. To assess the existence of dilated blood vessels in the tumor periphery correctly, we excluded lesions (1) without any endoscopic images at our hospital (12 lesions), (2) larger than 3 cm in diameter (184 lesions), and (3) without sufficient endoscopic images for judging the existence of dilated blood vessels by two experienced endoscopists (H.Y. and M.H.), who have each performed more than 5000 colonoscopies (165 lesions).

### 2.3 Endoscopic procedures

We used magnifying colonoscopies (PCF-Q 240ZI and PCF-Q260AZI, Olympus Co., Tokyo, Japan), attached to a processor (CV-260SL, Olympus Co.) and a light source (CLV-260SL, Olympus Co.) to assess the lesions in all cases. There were no adverse events in all cases.

### 2.4 Definitions

**Figure 1:**
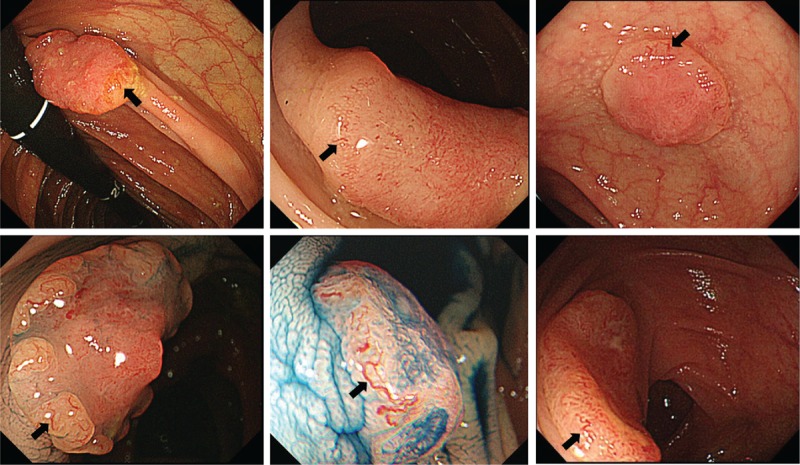
Dilated vessels (arrow) seen in the tumor periphery.

We defined the dilated blood vessels in the tumor periphery as the vessels thicker than those surrounding a type I pit that travels through two or more ducts over a type I pit (Fig. 1).

### 2.5 Evaluation

All lesions were classified into two groups according to the existence of dilated blood vessels in the tumor periphery. Furthermore, the gross type was classified as protruded (0-Ip, Isp, and Is), flat elevated (0-IIa), and depressed (0-IIc, IIa+IIc).^[10]^ Clinicopathological features, such as the depth of invasion, lymphovascular invasion (LVI), and poorly differentiated clusters (PDCs) were analyzed in all resected specimens based on the World Health Organization's criteria.^[11]^ We measured the submucosal invasion depth according to the guidelines issued in 2014 by the Japanese Society for Cancer of the Colon and Rectum for treating CRC.^[12]^ All lesions were independently assessed by two other experienced endoscopists (T.M. and H.H.), who have each performed more than 5000 colonoscopies. They were blinded to each pathological diagnosis, and they evaluated the existence of dilated blood vessels of the lesions from endoscopic images. When they did not agree, the existence of dilated blood vessels was regarded as negative.

### 2.6 Statistical methods

Data were analyzed by JMP Pro statistical software for Windows, version 11 (SAS, Tokyo, Japan). Continuous data were compared using unpaired Student's *t*-tests, whereas categorical variables were tested using Mann-Whitney *U* test. Values of *P* < 0.05 (two-tailed) were considered significant. The diagnostic accuracy was expressed as point estimates of accuracy rate with Wald-type 95% confidence interval (CI).

## 3 Results

**Figure 2:**
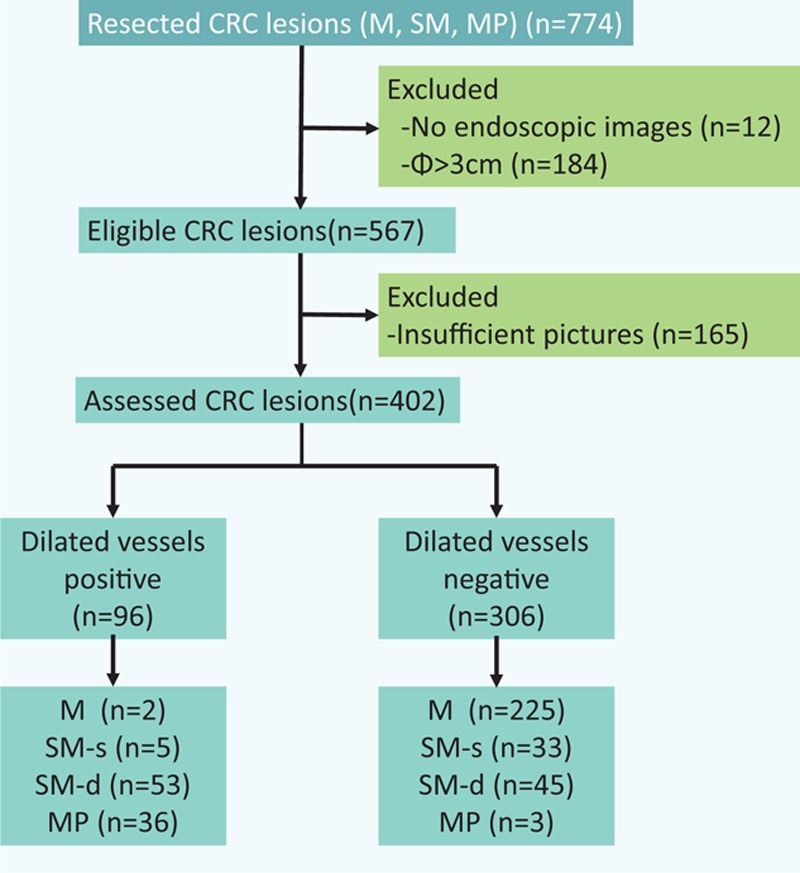
STARD diagram showing the number of enrolled lesions in this study.

A total of 402 consecutive small-sized CRC lesions in 380 patients were included. The invasion depth of the lesions was as follows: M 227, SM-s 38, SM-d 98, and MP 39. The dilated vessels were observed in 96 of 402 lesions (Fig. 2), but most of them were recognized in depressed lesions (Table 1). Thus, we evaluated the relationship between the existence of dilated vessels and age, location, invasion depth, and tumor size of the depressed lesion, and we found that the existence of dilated vessels and the tumor depth are closely related (Table 2). The existence of dilated vessels suggests SM invasion, especially SM-d or deeper invasion, in depressed lesions. Thus, the existence of dilated vessels may be a strong indicator of SM-d or deeper lesions (Table 3). The concordance rate between the two endoscopists was 98.2% (94/96 lesions), and the κ value was 0.93.

**Table 1:**
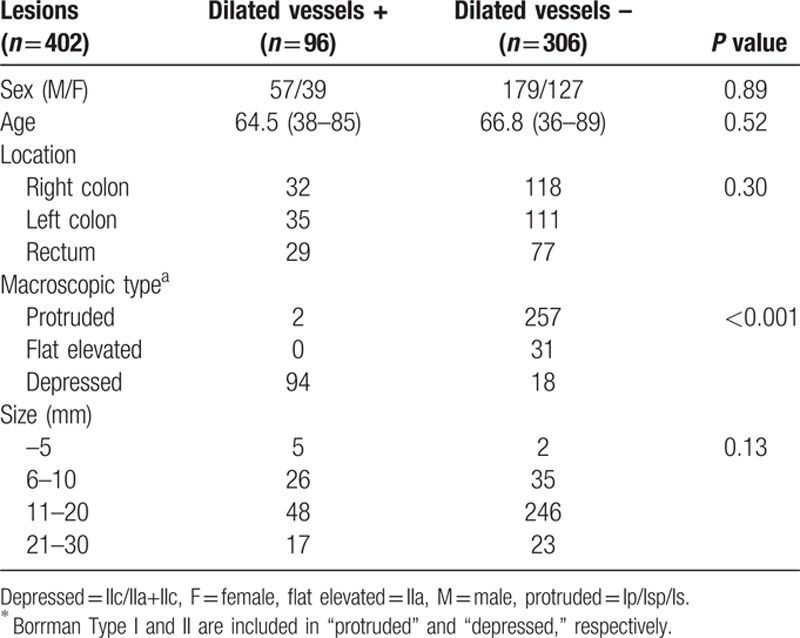
Correlation between the existence of dilated vessels and the clinical characteristics of 402 lesions with colorectal cancer.

**Table 2:**
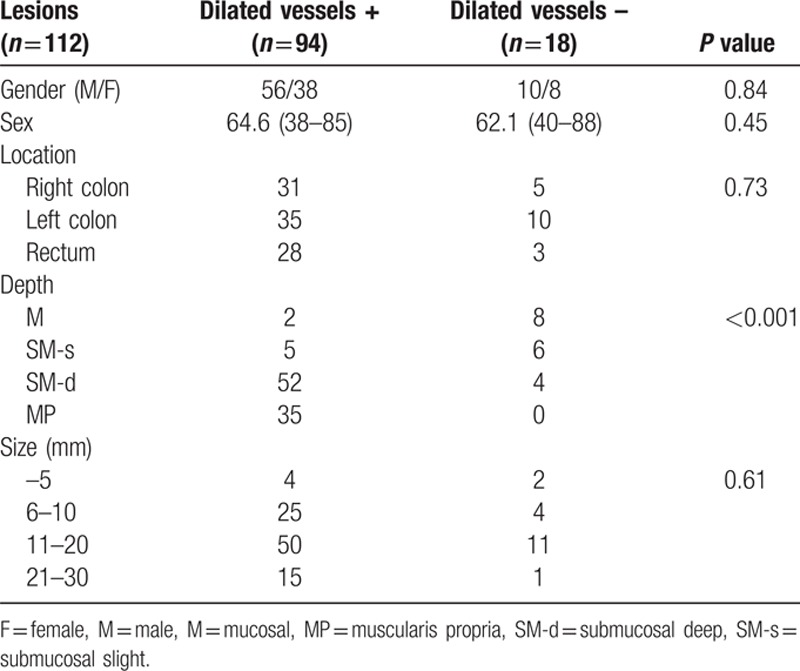
Correlation between the existence of dilated vessels and the clinical characteristics of 112 depressed lesions.

**Table 3:**
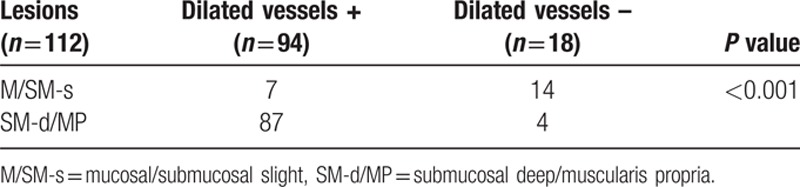
Correlation between the existence of dilated vessels and the invasion depth of depressed lesions.

When dilated vessels were used as an indicator of SM-d or deeper invasion in depressed lesions, the sensitivity was 95.6% (95% CI: 89.1, 98.8), specificity was 66.7% (43.0, 85.4), and accuracy was 90.2% (83.1, 95.0) (Table 4). The positive/negative likelihood ratio was 2.87/0.07 (range 1.56–5.26/0.02–0.18). The diagnostic odds ratio was 43.5 (range 11.3–168).

**Table 4:**
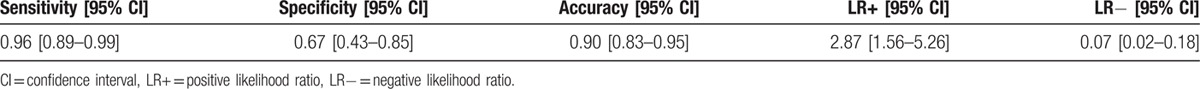
Sensitivity, specificity, and diagnostic accuracy of the dilated vessels for SM-d or deeper invasion in depressed lesions.

Histopathological analysis of the dilated vessels positive group showed submucosal cancer in 57 (59%) cases, LVI in 29 (51%), and PDC in 5 (8%). Similarly, the dilated vessels negative group had submucosal cancer in 79 (26%) cases, LVI in 33 (41%), and PDC in 12 (15%). Therefore, in relation to the existence of dilated vessels, LVI, and PDC, there were no significant differences between the groups (Table 5).

**Table 5:**
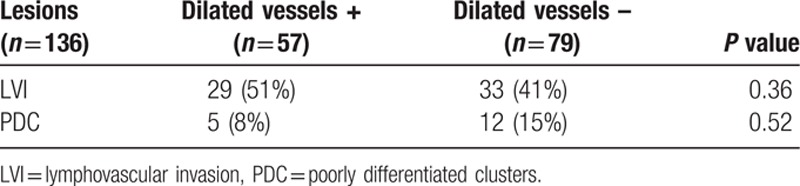
Correlation between the existence of dilated vessels and LVI or PDC in the lesions.

## 4 Discussion

Magnifying chromoendoscopy using pit pattern classification is considered the most accurate method for determining of the depth of invasion of early-stage CRC.^[4]^ The NBI classification can be easily used with or without magnifying endoscopy, and it has been advocated in recent years.^[5–7]^ Lately, narrow-band light observation with blue laser imaging magnification has been also used to determine the invasion depth of colorectal neoplasms and the diagnostic effectiveness of this method was similar to that of NBI magnification.^[13]^ Magnifying endoscopy requires close observation of the tumor surface's blood vessels and structure, which can sometimes be difficult to observe in real-world clinical settings due to bleeding or intestinal tract washing liquid residues. In addition, the diagnostic accuracy and inter-observer concordance of both classifications are not so high.^[14,15]^ The dilated vessels evaluated in the current study were easier to observe than the indicators that have been used thus far. The dilated vessels are also useful if obtaining a frontal internal view of the lesions is impossible or bowel preparation is insufficient.

Depressed lesions have a tendency to rapidly invade the submucosal layer, even when they are small. Oka et al reported that the prevalence of SM invasion in depressed lesions is about 20%, even in the case of a diminutive polyp (<5 mm), and about 50% in cases with polyps 6–10 mm.^[16]^ In the present study, there was no correlation between the tumor size and existence of dilated vessels in depressed lesions, although the existence of dilated vessels is a strong indicator of SM-d or deeper invasion. Thus, this information may help physician strongly suspect deep invasion in depressed lesions, even if the tumor size is small.

The origins of dilated blood vessels in the tumor periphery are unclear. Still, it is surmised that dilated vessels may originate from congestion of normal blood vessels rather than from tumors, as the blood vessel diameter in colorectal tumors is generally thicker in the central area and thinner in the tumor periphery.^[17]^

The present study did not include CRC with a diameter of over 30 mm because sometimes we cannot evaluate the entire circumference of the tumors due to their bigger size. Some studies have shown that most depressed lesions over 15 mm have SM-d or deeper invasion.^[18]^ In our study, there was no correlation between the tumor size and existence of dilated vessels in depressed lesions. Thus, we still consider dilated vessels an effective indicator, although this study did not include bigger lesions.

We recognize that there are some limitations in this study. First, this was an exploratory study performed at a single center and a desirable sample size was not calculated prior to the study. Therefore, we need to evaluate the effectiveness of the existence of the dilated vessels prospectively and compare it with the pit-pattern classification and Narrow-Band Imaging International Colorectal Endoscopic classification. Second, compared to the sensitivity, the specificity was insufficient in our study. The existence of dilated vessels is very useful for detecting SM-d or deeper invasion; however, if possible, endoscopists should perform magnifying chromoendoscopy to avoid overdiagnosis. Third, there could also be possible selection bias, as the subjects that were recruited only had malignant lesions. Additionally, many CRC lesions (165/742) were excluded because some endoscopists paid attention not to the periphery of the tumor, but to the center for pit-pattern diagnosis. However, our data suggest the possible usefulness of recognizing dilated vessels in the tumor periphery, which was not well understood until now.

In conclusion, the existence of dilated vessels in the tumor periphery suggests SM invasion, especially SM-d or deeper invasion, in depressed lesions. This information may help physicians decide treatment and encourage careful management of depressed lesions.
